# Ship Target Automatic Detection Based on Hypercomplex Flourier Transform Saliency Model in High Spatial Resolution Remote-Sensing Images

**DOI:** 10.3390/s20092536

**Published:** 2020-04-29

**Authors:** Jian He, Yongfei Guo, Hangfei Yuan

**Affiliations:** 1 Changchun Institute of Optics, Fine Mechanics and Physics, Chinese Academy of Sciences, Changchun 130033, China; guoyf@ciomp.ac.cn (Y.G.); yuanhangfei@ciomp.ac.cn (H.Y.); 2 University of Chinese Academy of Sciences, Beijing 100049, China

**Keywords:** HFT, ship detection, remote sensing, saliency model, ResNet

## Abstract

Efficient ship detection is essential to the strategies of commerce and military. However, traditional ship detection methods have low detection efficiency and poor reliability due to uncertain conditions of the sea surface, such as the atmosphere, illumination, clouds and islands. Hence, in this study, a novel ship target automatic detection system based on a modified hypercomplex Flourier transform (MHFT) saliency model is proposed for spatial resolution of remote-sensing images. The method first utilizes visual saliency theory to effectively suppress sea surface interference. Then we use OTSU methods to extract regions of interest. After obtaining the candidate ship target regions, we get the candidate target using a method of ship target recognition based on ResNet framework. This method has better accuracy and better performance for the recognition of ship targets than other methods. The experimental results show that the proposed method not only accurately and effectively recognizes ship targets, but also is suitable for spatial resolution of remote-sensing images with complex backgrounds.

## 1. Introduction

With the development of remote-sensing technology, the quality of remote-sensing images acquired has become very high, while the high spatial-resolution remote-sensing images are increasingly used in various fields [1,2]. Therefore, it is also very useful to achieve important strategic target recognition on remote-sensing images (e.g., ships, airports, aircraft, etc.) [3,4]. However, the general target recognition algorithm is not suitable for use in remote-sensing images, Hence, in order to recognize meaningful targets in spatial resolution remote-sensing images, this study proposes a novel target recognition algorithm.

Ships are important targets for monitoring, emergency rescue, etc. The detection and identification of ships can monitor the distribution of ships in key sea areas and can also meet the actual work needs of maritime traffic control, maritime search and rescue. However, if ship recognition depends only on manual processing, there will be many problems, such as heavy workload, low efficiency, high repetitiveness, strong subjectivity, high cost, etc., that cannot meet the efficient information needs of modern society. Therefore, ship detection and recognition is a research hotspot in the field of image recognition [5]. In general, traditional remote sensing image ship detection methods are based on gray threshold segmentation and grayscale statistics [6,7,8]. These methods are suitable for uniform sea surface textures, low water surface gray value and high contrast between the sea surface and ships. However, in some complicated situations, such as the occurrence of waves, cloud cover and low gray value of the ship, it is easy for false alarms to appear. Another popular method is based on deep learning [9,10]. This type method has higher detection accuracy, but it requires preparing templates in advance; there also are problems of relying on prior knowledge and high training complexity. Other commonly used methods are edge detection [11] and fractal models [12]. The former uses the hull to find obvious edge features on the image. Using this, this method can extract the edge contour and judge whether it is a ship, according to its shape characteristics. The latter uses contrasting classification characteristics of natural and artificial background s for detection, but detection error is higher under the influence of cloud and fog.

Methods based on visual saliency can quickly find targets of interest in complex scenes, so they have become a research hotspot for ship detection in recent years [13,14]. At present, saliency detection methods are mainly divided into methods based on spatial domain models and methods based on frequency domain models. The spatial domain models mainly include ITTI model [15], attention based on information maximization (AIM) model [16], graph-based visual saliency (GBVS) model [17], context aware (CA) model [18], frequency-tuned saliency (FT) model [19], histogram contrast (HC) model [20], etc. These types of models achieve target detection by fusing and extracting multiple features of the image. However, in remote sensing images, the ship target size is small, so environmental factors such as sea surface texture, weather, illumination, etc. easily interfere with the feature extraction process. Therefore, spatial domain models not only have poor background suppression ability, but also take large processing times. Conversely, frequency domain-based saliency detection methods have significant advantages in terms of background suppression and computational speed. Hou proposed a spectral residual (SR) method [21], which is an early saliency model based on frequency domain analyses. From the perspective of information theory, this method removes the background information from the global range of the image in the frequency domain and retains saliency information—which is the saliency region corresponding to the spatial domain. In order to handle the multi-channel features of color images, Guo proposed a phase quaternion Fourier transform (PQFT) method [22], which has good edge detection performance. However, targets detected by this method have poor internal continuity. Li proposed a theory of hypercomplex Flourier transform (HFT) method [23,24], which has a better effect on target integrity detection. However, this method is not strong in background suppression and has weak ability to distinguish multiple targets at close distance.

In view of the above problems, this study proposes a ship detection and recognition method based on the MHFT saliency model. This method includes three parts: ship target detection, regions of interest (ROIs) extraction and ship target recognition. The ship target detection part obtains the candidate ship area by using the MHFT method, which can more effectively suppress the sea surface, cloud and sea surface texture interference, enhance target integrity detection and the ability to distinguish between targets, thus improving the detection ability of small targets. In the ROIs extraction part, we use the OTSU method to extract the regions of interest, which can effectively extract the saliency region in the saliency map, segment the target regions of the candidate ship target, and then realize the recognition of these segmented regions. In the ship target recognition part, we use a method of ship target recognition based on ResNet framework [25]. By combining with the transfer learning model, we can achieve higher classification accuracy with less training data.

The rest of the study is organized as follows: In Section 2, we introduce the preprocessing method, the MHFT method, the methods of ship target candidate extraction and the method of ship target recognition. The results of ship target recognition are shown in Section 3. The conclusion is provided in Section 4.

## 2. Materials and Methods

### 2.1. MHFT Saliency Model

Saliency analysis is an effective technique for detecting ROIs because it can rapidly and accurately divide an image into foreground targets and background regions [26]. Various saliency analysis models have emerged. Among them, the frequency domain-based saliency detection models perform best because the frequency domain characteristics of natural images have scale invariance. Hou proposed a simple and fast SR method for calculating the saliency of single-channel grayscale images based on the above theory [21]. Then, Li proposed the HFT method to extend the SR method to detect saliency objects in the color image [24]. In essence, this method analyzes the manifestation and difference of the saliency target and background regions in the frequency domain and extracts the saliency regions with the frequency domain multi-scale method by combining with the hypercomplex Fourier transform, thus obtains a better detection effect. This method uses luminance I, red–green C_RG_ and blue–yellow C_BY_ to construct quaternion images, and then makes discrete Flourier transform of quaternion images. Finally, the method performs multi-scale Gaussian smoothing on the amplitude spectrum in the frequency domain to obtain multi-scale saliency maps. Hence, we can choose the best saliency map from these saliency maps. However, the HFT model is similar to other frequency-domain saliency models that lack the theoretical basis of biologic vision, resulting in incomplete removal of sea clutter and other disturbances. In addition, since the HFT model uses a fixed-scale Gaussian kernel function to calculate the saliency map, there is no target change scale for different sizes. Hence, the target regions will be enlarged or incomplete when the saliency regions are extracted. Similarly, the HFT model has some defects in the calculation of the saliency map and the determination of the final saliency map. Therefore, this study has improved the HFT method in the aspects of color space selection, scale selection, saliency map calculation and final saliency map generation, which reduces the above shortcomings and obtains a more effective saliency map result. Next, we introduce the MHFT method in detail.

#### 2.1.1. Color Space Selection

The most commonly used color space in image representation is the RGB color space. However, the role of the RGB color space is to better display color images without considering human visual perception. Hence, it is not suitable for calculation of saliency detection. On the contrary, CIE Lab color space not only contains the entire gamut of RGB color space [27], but also can show more colors that can be perceived by human eyes. Hence, the CIE Lab space is closer to the human visual system (HVS) and can compensate for the unevenness of color distribution in the RGB color space. Based on the above reasons, this study uses CIE Lab color space instead of RGB color space to improve HFT model.

#### 2.1.2. Hypercomplex Fourier Transform Model

The hypercomplex Fourier transform has been widely used in color image processing [28]. In general, the hypercomplex matrix is specified as a quaternion; the quaternion can be used to combine the different features of the image so that image can be represented as quaternion. The expression of the hypercomplex number or quaternion matrix of the image is shown in Equation (1):

(1) $$f(x,y)=w_{1}f_{1}+w_{2}f_{2}i+w_{3}f_{3}j+w_{4}f_{4}k$$

where $w_{1}\sim w_{4}$ are weights; $f_{1}\sim f_{4}$ are feature maps, $f_{1}$ is motion feature, $f_{2}$ is luminance feature, $f_{3}$ and $f_{4}$ are color features. For static remote sensing images, $f_{1}=1$, $f_{2}\sim f_{4}$ are defined as Equations (2)–(4):

(2) $$f_{2}=(r+g+b)/3$$

(3) $$f_{3}=R-G$$

(4) $$f_{4}=B-Y$$

where *r*, *g* and *b* represent the red, green and blue channels of the input color image; R=r−(g+b)/2; G=g−(r+b)/2; B=b−(r+g)/2; Y=(r+g)/2−|r−g|/2−b; $w_{1}=0$; $w_{2}=0.5$; $w_{3}=w_{4}=0.25$.

In the CIE Lab color space, it is necessary to modify the HFT model to make it more consistent with the HVS. First, we replace the $f_{2}\sim f_{4}$ feature maps with *L*, *a* and *b* that remove the low frequency information, so the improved three feature maps are shown in Equations (5)–(7):

(5) $$F_{2}=L-L_{1}$$

(6) $$F_{3}=a-a_{1}$$

(7) $$F_{4}=b-b_{1}$$

where $F_{2}^{}$, $F_{3}$ and $F_{4}$ are used instead of $f_{2}\sim f_{4}$ respectively; $L_{1}$, $a_{1}$ and $b_{1}$ are the mean values of each channel over the whole image. Then we take the Fourier transform of the quaternion and get its polar representation:

(8) $$F[u,v]=\left\|F[u,v]\right\|e^{\mu \Phi (u,v)}$$

where F[u,v] is the Fourier transform of f(x,y) and the definition of the amplitude spectrum, phase spectrum and pure quaternion matrix are as follow:

(9) A(u,v)=‖F(u,v)‖

(10) $$P(u,v)=\Phi (u,v)=\tan^{-1}\frac{\left\|v(F(u,v))\right\|}{S(F(u,v))}$$

(11) $$\chi (u,v)=\mu (u,v)=\frac{v(F(u,v))}{\left\|v(F(u,v))\right\|}$$

The results of classical HFT model show that the amplitude spectrum contains both salient and non-salient information. Therefore, HFT adopts different Gaussian kernel functions to smooth and filter the amplitude spectrum so as to suppress the high frequency information and enhance the low frequency information. After smooth filtering of different scales, a spectral scale space is obtained. The definition of Gaussian kernel function and spectral scale space are shown in Equations (12) and (13):

(12) $$g(u,v;k)=\frac{1}{\sqrt{2\pi }2^{k-1}t_{0}}e^{-(u^{2}+v^{2})/(2^{2k-1}t_{0}^{2})}$$

(13) Λ(u,v;k)=(g(.,.;k)A)(u,v)

where *k* is the spatial scale parameter, k=1,2,…,K−1,K, K is determined by the size of the image, $K=[\log_{10}\min\{H,W\}]+1$, *H* and *W* represent the height and width of the image, respectively. By smoothing the amplitude spectrum, we can obtain the saliency of different scales $s_{k}$:

(14) $$s_{k}=g*{\left\|F_{H}^{-1}\{\Lambda (u,v)e^{\chi P(u,v)}\right\|}^{2}$$

However, Equation (14) uses Gaussian function of fixed scale to generate saliency map, which is not conducive to the display of salient of regions of different sizes. Therefore, we propose an adaptive scale Gaussian fuzzy model based on actual saliency regions size of the image. In this model, the larger scale Gaussian fuzzy kernel function is used to deal with the smaller scale saliency map and the smaller scale Gaussian fuzzy function is used to deal with the larger scale saliency map, according to this principle, we get the adaptive Gaussian fuzzy kernel function:

(15) $$G(u,v;k)=\frac{1}{\sqrt{2\pi }\mu \sigma }e^{-(u^{2}+v^{2})/2{\left(\mu \sigma \right)}^{2}}$$

where *k* is the scale; μ is the adaptive and it’s a linear function of *k*, which is defined as Equation (16); σ=W∗0.04, W is the width of the image.

(16) $$\mu =1.5-\frac{1}{8}k$$

Lastly, we obtain the final saliency map sequence:

(17) $$s_{k}=G*{\left\|F_{H}^{-1}\{\Lambda (u,v)e^{\chi P(u,v)}\right\|}^{2}$$

Then we need to determine the final saliency map from a series of saliency maps {$s_{k}$}. In the classical HFT model, it directly regards the saliency map with the minimum entropy as the optimal saliency map and others are abandoned. However, in our experiment, we found that the minimum entropy saliency map sometimes does not contain the complete information, which may lead to inaccurate detection. For all the above reasons, the spatial contrast function (SCF) is taken for selecting the optimal saliency map.

In the theory of statistics, a probability density function (PDF) can describe the relative likelihood of values [29]. Hence, after the normalization of k saliency maps, they can be regarded as the PDF:

(18) $$p_{k}(x,y)=\frac{S_{k}(x,y)}{\sum_{x,y}S_{k}(x,y)},k=1,2,\ldots ,K$$

where $S_{k}(x,y)$ is the *k*th saliency map. Subsequently, standard deviation of the bivariate distribution can be defined as:

(19) $$\sigma_{k}=\sqrt{\sum_{x,y}\left({\left(x-E_{kx}\right)}^{2}+{\left(y-E_{ky}\right)}^{2})p_{k}(x,y\right)}$$

where $E_{kx}=\sum_{x,y}xp_{k}(x,y)$ and $E_{ky}=\sum_{x,y}yp_{k}(x,y)$ denote expectation values of a saliency map $S_{k}(x,y)$ in x and y coordinates, respectively. Since the standard deviation of the image reflects the degree of aggregation of the spatial distribution, the selected saliency maps should be within a smaller standard deviation range. Hence, in this study, saliency maps with standard deviation less than $\alpha \sigma_{\min}$ are selected as candidate saliency maps, where $\sigma_{\min}$ denotes the minimum standard deviation. Optimal performance can be achieved when α is set to 1.5 in this study.

Then we use the contrast function to select the optimal saliency map from candidate saliency maps:

(20) $$C_{k}=\frac{\sum_{(x,y)\in R_{k}}s_{k}(x,y)}{\sum s_{k}(x,y)}$$

where $R_{k}$ denotes the potential salient regions in saliency map $s_{k}(x,y)$. The narrower the potential salient region, the smaller the value of the contrast function. Therefore, the selected optimal saliency map $S_{final}$ should have a greatest contrast function value.

#### 2.1.3. Experimental Results

**Figure 1:**
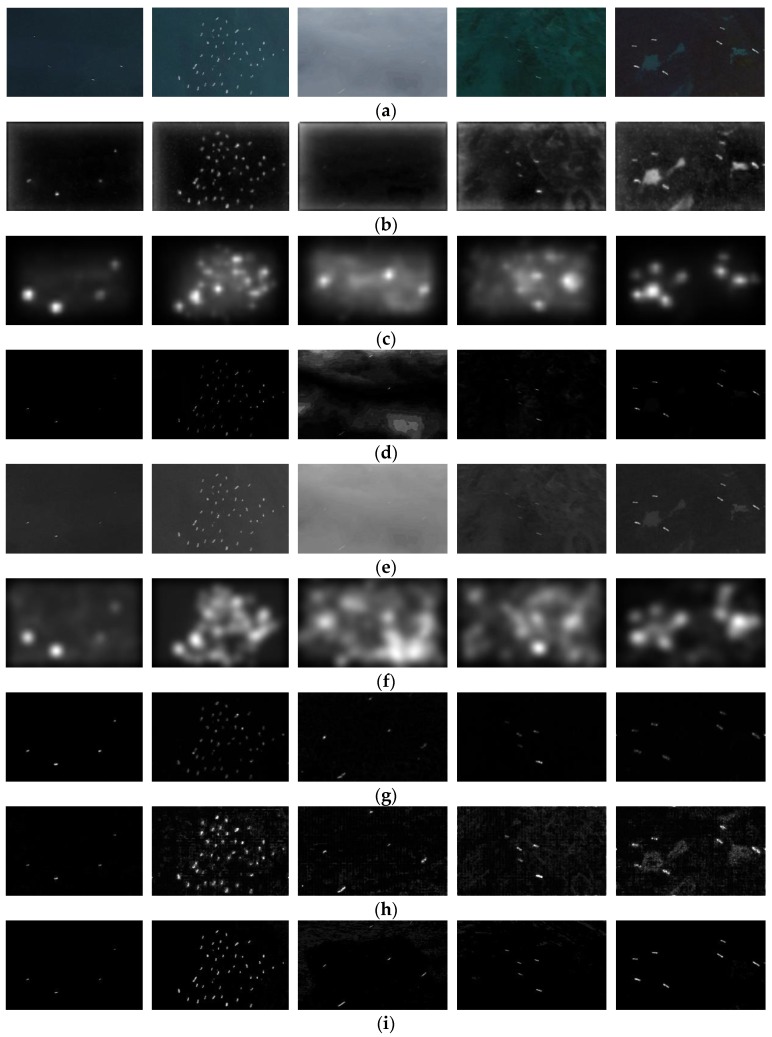
Comparison of several saliency detection method: (**a**) original images; (**b**) ITTI; (**c**) graph-based visual saliency (GBVS); (**d**) spectral residual (SR); (**e**) histogram contrast (HC); (**f**) Principal Component Analysis (PCA); (**g**) phase quaternion Fourier transform (PQFT); (**h**) hypercomplex Flourier transform (HFT); (**i**) proposed method.

In order to evaluate the experimental results of saliency detection, we analyzed them from qualitative and quantitative aspects. In qualitative experiments, we used the Google Earth dataset to compare ITTI, GBVS, SR, HC, PCA, PQFT, HFT and our model. Some representative qualitative experiment results are shown in Figure 1. The experiments are carried out on a computer: Image resolution, 756 × 493; Image number: 60; Intel i5 3.5 GHz; RAM 16 G, GTX 1080.

As show in Figure 1, the spatial domain saliency detection method has weak suppression ability for the sea surface background such as the sea cloud and sea waves (such as Figure 1b,c), while the frequency domain detection method has better suppression ability (such as Figure 1d,g), but the saliency object regions obtained by SR and PQFT have obvious detection incompleteness and it is not imperfect to remove cloud interference. HC can detect complete saliency objects, but it filters out little background information. The detection result of PCA is relatively poor, not only does it fail to effectively filter out the background information, but the detected saliency objects have expanded a certain number of pixels, resulting in the phenomenon of target aliasing. HFT can basically detect complete saliency objects, but it still contains certain redundant background information. Based on the classic HFT method, improved components of CIE Lab color space used in this study are more line with the human visual perception. Moreover, the adaptive scale Gaussian fuzzy model can achieve clear inspection of saliency regions of different sizes. By selecting the optimal saliency map through spatial standard deviations and contrast function, we can highlight both boundaries and their internal regions of saliency objects. As compared with HFT, the proposed method can accurately detect complete saliency objects in the presence of complex texture background information, as show in Figure 1i.

**Figure 2:**
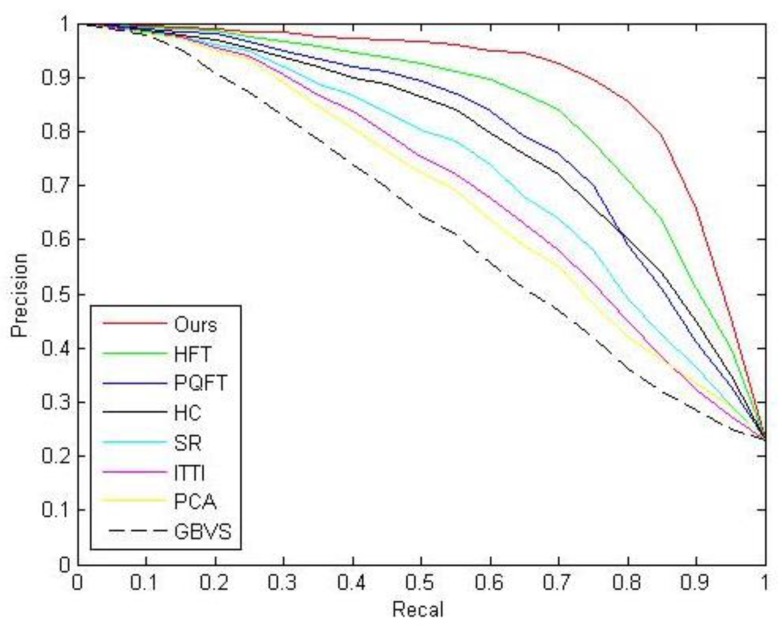
Precision–recall curves.

To verify further the performance of our proposed method, we carry out a quantitative analysis on it. We use the PR curve to evaluate the performance of the proposed method, its horizontal and vertical coordinate are the precision (P) and the recall (R), they are defined as follows:

(21) $$P=\frac{A\cap B}{B}=\frac{\sum_{(x,y)}A(x,y)B(x,y)}{\sum_{\left(x,y\right)}B(x,y)}\times 100\%$$

(22) $$R=\frac{A\cap B}{A}=\frac{\sum_{\left(x,y\right)}A(x,y)B(x,y)}{\sum_{\left(x,y\right)}A(x,y)}\times 100\%$$

(23) $$F=\frac{(1+\alpha )\times P\times R}{\alpha \times P+R}\times 100\%$$

where A(x,y) denotes the ground-truth images; B(x,y) denotes the binary images corresponding to the saliency maps; F-Measure (*F*) is the comprehensive evaluation index of *P* and *R*, in this experiment, when α=1, *F* combines the results of *P* and *R*. We use *Acc* to evaluate the accuracy of ship detection in this study, it is defined as follows:

(24) $$Acc=\frac{CR}{SUM}\times 100\%$$

where SUM denotes the total number of ships in the image, CR represents the number of ships correctly detected. According to the above formula, we calculated the values of *P*, *R* and *Acc* as shown in Table 1, for the proposed method, we are pursuing high *P*, *R* and *Acc*. Figure 2 shows a comparison of PR curves of the proposed method and other methods, we can evaluate the performance of the methods by comparing the area under the curve of these methods, the larger area under the curve, the better performance of the method. According to Figure 2 and Table 1, our method performs better than other methods.

### 2.2. Recognition of Ship Types

**Figure 3:**
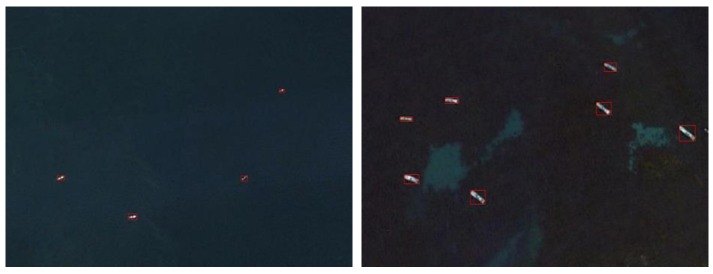
Ship area extraction results.

After obtaining the saliency map, we need to segment the saliency map to extract the candidate regions of the ship. Because of the contrast between the target and the background is very obvious, this study uses OTSU method to extract the region of interest. The specific formula is:

(25) $$S(x,y)=\{\begin{array}{c} 0,\;\;\;\;\;\;\;\;\;\;\;\;\;\;\;\;\;S(x,y)<T\\ 1,\;\;\;\;\;\;\;\;\;\;\;\;\;\;\;\;\;S(x,y)>T \end{array}$$

where T is the adaptive segmentation threshold calculated by the OTSU method. After thresholding the saliency map, an eight-connected region method is used to split in order for the achievement of the target region to be detected and calculate the region of the rectangle. In order to ensure the continuity of hull, each partition of the region must be detected for calculating the maximum length and the width, in order to secure the region without losing part of ship target, on the base of the aspect to expand pixels. This results in a rectangular region containing a specific length and width of the target region to be detected, as shown in Figure 3. After extracting the regions of interest, we make a simple judgment on these regions to remove some obvious pseudo-ship targets. In this study, regions of interest with a side length of less than 5 pixels and a side length of more than 150 pixels are considered as noise and pseudo-regions of interest with a large region of land and large islands.

After obtaining the saliency regions, we realize the recognition of the candidate ship target. The traditional target recognition methods (such as SVM [30]) have a good effect on two-class classification with obvious features due to the simple model. However, for small and complex targets, the features of traditional recognition methods make it difficult to exactly describe the target objects, which makes it difficult for the experimental results to reach sufficient accuracy. In recent years, deep learning models have shown good performance in various applications such as target classification, scene recognition and target detection. Among them, convolutional neural network (CNN) [31] has strong universality under the supervision of large amounts of data and it has certain robustness to object deformation, background interference and light changes. Therefore, this study proposes a residual convolutional neural network model (ResNet) based on transfer training to classify and recognize saliency objects.

**Figure 4:**
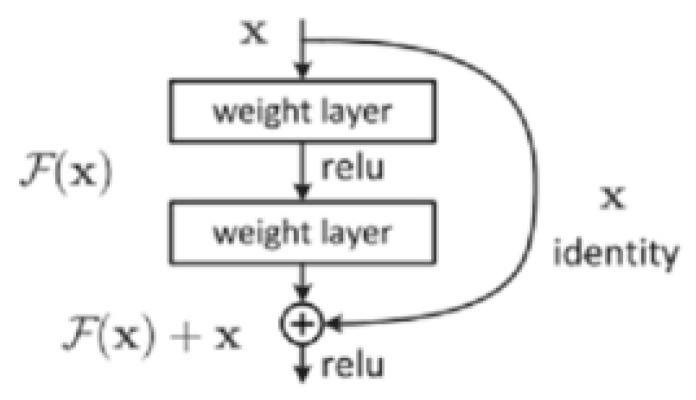
Residual principle.

**Figure 5:**
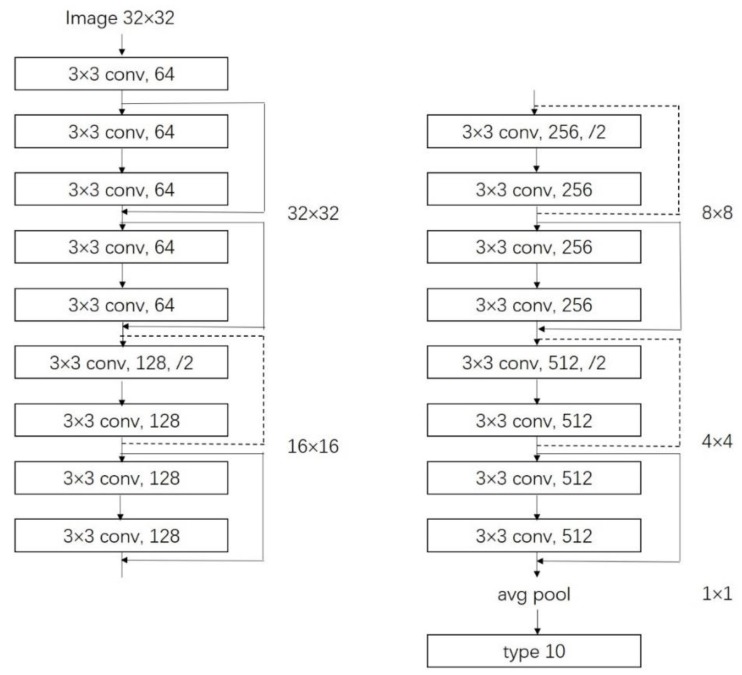
ResNet for transferring.

The number of CNN layers greatly affects the performance of feature extraction when the number of layers in the network reaches a certain degree. Not only is the training process more complicated, but also problems of gradient disappearance, gradient degradation and gradient explosion will occur, resulting in difficulty in improving the accuracy of the CNN model. He et al. introduced a residual mechanism in the CNN model [25]. This method adds cross-layer connectivity and identity mapping mechanisms to the network model. As shown in Figure 4, X is the input image, $F=\omega_{2}\varphi (\omega_{1}X)$ denotes the output after two layers of convolution, then the final output of each residual block is y=ωX+F, φ denotes activation function, ω is a nonlinear mapping to ensure that the dimensions of the out are the same. The ResNet model solves the problems of complex training and gradient degradation when the number of network layers deepens, making its model performance distinctly better than other models and becoming one of the most accurate and widely used CNN models. Therefore, we choose the ResNet model as our saliency objects recognition model. As shown in Figure 5, the input image size of the ResNet model used in this study is 32×32.

**Figure 6:**
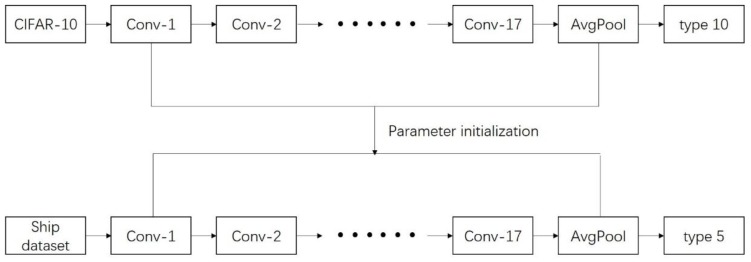
Transferring learning.

In order to use a sufficient number of training samples, we adopt the method of transfer training. Transfer learning is a deep learning technique that is very suitable for small amounts of data. The principle is to use the existing dataset to train the proposed model, and then make it suitable for our problem through simple adjustments. We used a pre-training ResNet model and a CIFAR-10 dataset to achieve the transfer learning training [32], and then fine-tune the trained parameters as initialization parameters, making this model suitable for our ship recognition method, as shown in Figure 6.

**Figure 7:**
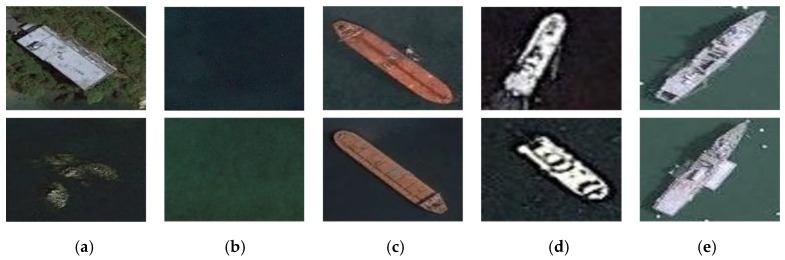
Samples of our dataset: (**a**) land, (**b**) marine, (**c**) cargo ship, (**d**) steamer, (**e**) warship.

The ResNet model we used was the pre-training model provided by PyTorch; The CIFAR-10 [33] dataset contains 10 categories. Each category has 6000 color images of size 32×32, so the data volume is sufficient to train the proposed ResNet model. Then we use the ship dataset to fine-tune the proposed model. For the specific application needs of ship target recognition, the samples collected in this study include five categories of marine, land, cargo ship, steamer and warship for model training and testing, as shown in Figure 7. We selected 300 images in each category, of which 250 images were used as training samples and 50 images were used as test samples. The training samples were expanded to 4 times the original through image rotation. We choose a variety situations for the selection of the sample: the choice of the sea surface included the situation of cloud cover; the choice of the ship includes various shapes; the land image was added to identify the offshore land and islands. The parameters trained on the CIFAR-10 dataset were used as initial parameters and it was trained on the ship dataset with a lower learning rate. Some of the parameters are shown in Table 2. After training, the accuracy rate on the training dataset was stable at 98.15% and the accuracy rate on the test dataset was stable at 95.45%. The results show that the proposed model had a high classification accuracy and can effectively distinguish various types of ships.

## 3. Results

When the ResNet model training was completed, we performed unsupervised recognition on the proposed model and compared it with the recognition results of the HOG+SVM [30,34], AlexNet, GoogleNet and VGG16. We selected a total of 1210 images, including 250 sea surface images, 250 land images, 280 cargo ship images, 280 steamer images and 150 warship images. We used Receiver Operating Characteristic (ROC) curve and PR curve to evaluate the experiment results. Unlike the saliency detection experiment, we needed to redefine the horizontal and vertical coordinates of the curve.

**Figure 8:**
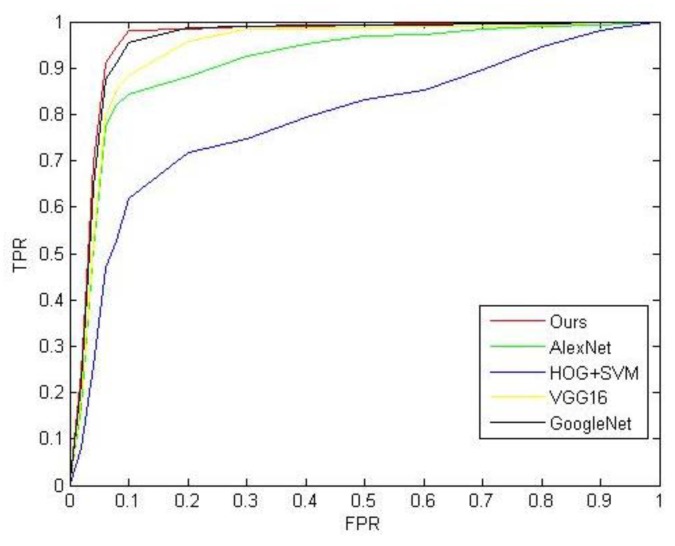
ROC curve.

In the ROC curve, we used TPR and FPR as the horizontal and vertical coordinates, they were defined as follows:

(26) $$TPR=\frac{TP}{TP+FN}\times 100\%$$

(27) $$FPR=\frac{FP}{TN+FP}\times 100\%$$

where TP denotes the number of true positive ship recognition targets, TN denotes the number of true negative ship recognition targets, FP denotes the number of false positive ship recognition targets, FN denotes the number of false negative ship recognition targets. According to the above formula, we calculated the values of TPR and FPR as shown in Table 3. Figure 8 shows a comparison of ROC curves of the proposed model and other models. We can evaluate the performance of the models by comparing the area under the curve: the larger area under the curve, the better performance of the model. According to Figure 8 and Table 3, our model performed better than other models.

**Figure 9:**
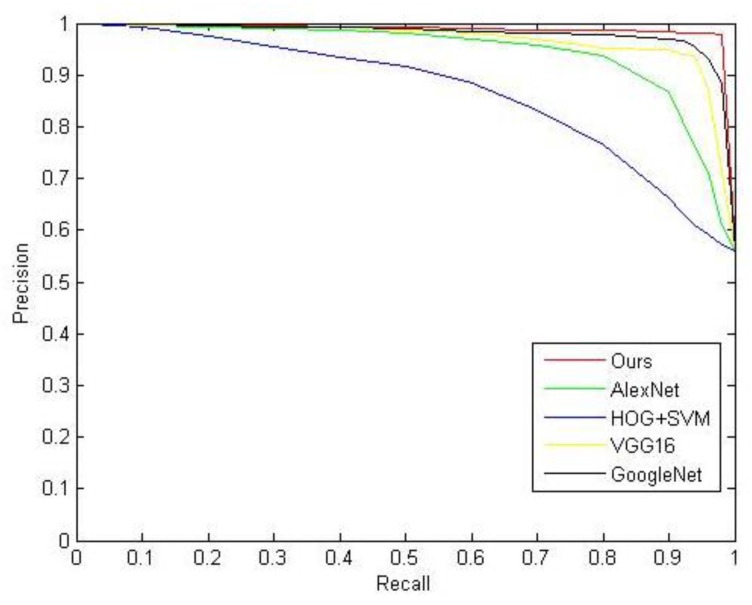
Precision–recall curves for recognition model.

In the PR curve, we used Recall and Precision as the horizontal and vertical coordinates. They are defined as follows:

(28) $$P=\frac{TP}{TP+FP}\times 100\%$$

(29) $$R=\frac{TP}{TP+FN}\times 100\%$$

(30) $$F=\frac{(1+\beta )\times P\times R}{\beta \times P+R}\times 100\%$$

where F-Measure (*F*) is the comprehensive evaluation index of *P* and *R*. In this experiment, β=1; the remaining variables are defined as shown previously. Similarly, we calculated the values of *P* and *R* as shown in Table 4. For the proposed model, we are pursuing high *P* and *R*. Figure 9 shows a comparison of PR curves of the proposed model and other models. We can evaluate the performance of the models by comparing the area under the curve of these models: the larger area under the curve, the better performance of the model. According to Figure 9 and Table 4, our model performed better than other models.

In summary, this study uses the ResNet model based on transfer learning to recognize saliency objects, the time consumption of each stage is shown in Table 5. As a comparative experiment, we used a method combining saliency detection and target classification to compare the time consumption of the proposed method [35]. The results showed the proposed method achieved the expected consequences in terms of recognition accuracy and recognition rate, which met the application requirements.

## 4. Conclusions

In this study, we proposed a novel ship target automatic detection and recognition based on HFT saliency methods for spatial resolution remote-sensing images. In the ship saliency detection section, we improved the HFT model in color space selection, scale selection, saliency map calculation and final saliency map generation, which can effectively remove background interference such as clouds and sea surface textures. Then we used the OTSU method to extract the regions of interest. After obtaining the candidate ship target regions, we used the ResNet model based on transfer learning for needs of the experiment, which not only solved the problem of gradient degradation caused by deepening the number of CNN model layers, but also made it possible to train a CNN model with a small amount of data and achieve higher classification of ship targets. In the experiment, the method in this study was compared with other classic methods in qualitative and quantitative evaluation. The experimental results show that the proposed method effectively overcomes the interference of the sea surface background and achieved high-precision and rapid detection and recognition of ship targets in complex background. The proposed method may be extended to other man-made target extraction in high spatial resolution remote-sensing images, such as airport and military base, which requires additional research.

## Figures and Tables

**Table 1:** Comparison of related numerical indicators for different methods.

Method	Precision	Recall	F-measure	Acc
ITTI	80.72%	42.17%	55.40%	54.17%
GBVS	74.14%	38.45%	50.64%	50.36%
SR	83.25%	43.59%	57.56%	59.38%
HC	80.46%	59.14%	68.17%	71.42%
PCA	77.42%	44.15%	56.23%	52.47%
PQFT	84.12%	55.34%	66.76%	68.17%
HFT	85.58%	68.49%	76.09%	73.45%
Ours	91.42%	72.47%	80.85%	82.36%

**Table 2:** Partial training parameters.

Sample Data	Learning Rate	Decay	Steps	Momentum	Batch Size
CIFAR-10	0.001	0.0001	120,000	0.9	128
Ship dataset	0.0001	0.0001	52,000	0.9	128

**Table 3:** TPR, FPR of our method and competing methods.

Method	TPR	FPR
HOG + SVM	89.86%	70.60%
AlexNet	91.81%	25.40%
VGG16	95.77%	19.60%
GoogleNet	97.75%	14.20%
Our model	98.45%	11.40%

**Table 4:** Comparison of related numerical indicators for different methods.

Method	Precision	Recall	F-Measure
HOG + SVM	64.38%	89.86%	75.02%
AlexNet	83.70%	91.81%	87.57%
VGG16	87.40%	95.77%	91.39%
GoogleNet	90.72%	97.75%	94.10%
Our model	92.46%	98.45%	95.36%

**Table 5:** Running times of each stage.

Method	Saliency Detection(s)	Recognition Model(s)
Literature [35]	0.253	0.835
Proposed method	0.237	0.047
